# Factors associated with antimicrobial drug prescription among inpatient dogs and cats at an academic veterinary hospital

**DOI:** 10.1017/ash.2022.186

**Published:** 2022-05-16

**Authors:** Emma Price, Claire Fellman, Annie Wayne, Manlik Kwong, Kirthana Beaulac, Shira Doron

## Abstract

**Background:** Widespread antimicrobial use in dogs and cats drives antimicrobial resistance in both animals and humans. Knowledge of the factors associated with antimicrobial use is limited in veterinary medicine. We examined factors associated with antimicrobial drug prescription among inpatient dogs and cats at an academic veterinary hospital. **Methods:** A veterinary-adapted observational medical outcomes partnership common data model was utilized to extract demographic, clinical, and prescription data from the electronic medical record system in this descriptive observational study. Using generalized estimating equations, we assessed the association between demographic and clinical factors and systemic antimicrobial drug prescription among inpatient dogs and cats at a small-animal teaching hospital between 2018 and 2020. **Results:** Across 11,685 dogs with 14,328 admissions (mean age, 7.4 years; 47% females), the following factors were associated with increased odds of any antimicrobial drug prescription: female, longer admission, a history of chemotherapy within 30 days of hospital admission, surgery upon admission or within the last 30 days, urinary catheterization, ICU admission, and oxygen support. In 3,371 cats with 4,088 admissions (mean age, 8.6 years; 39% females), the following factors were associated with increased odds of any antimicrobial drug prescription: female, longer admission, increased age (>8 years), admission into the ICU, surgery upon admission, and feline that did not require oxygen support or urinary catheterization. **Conclusions:** This study identifies multiple patient and clinical factors associated with increased risk of antimicrobial drug use in inpatient dogs and cats that can inform veterinary antimicrobial stewardship efforts and may be useful for antimicrobial use benchmarking on an institutional or multi-institutional scale.

**Funding:** None

**Disclosures:** None

